# An evaluation of temporal changes in physicochemical properties of gully pot sediments

**DOI:** 10.1007/s11356-022-20341-8

**Published:** 2022-04-29

**Authors:** Haoyu Wei, Tone Merete Muthanna, Lian Lundy, Maria Viklander

**Affiliations:** 1 Department of Civil, Environmental and Natural Resources Engineering, Luleå University of Technology, 97187 Luleå, Sweden; 2 Department of Civil and Environmental Engineering, Norwegian University of Science and Technology, NO-7491 Trondheim, Norway

**Keywords:** Sediment, Gully pot, Catchment management practices, Heavy metals, Solids accumulation rate, Particle size

## Abstract

Diffuse pollution is recognised as a major challenge in achieving EU Water Framework Directive compliance, with urban runoff being a key pathway connecting various sources to receiving waters. Gully pots, as one of the ubiquitous urban drainage infrastructures, are placed at the inlets of piped drainage pipe network and actively drain runoff from urban catchment with suspended solids proportionally retained. The physiochemical properties of these retained solids reflect the activities within the catchment during the accumulation period. In this work, seven gully pots in two catchment types (highway and housing) in Luleå, Sweden were fully emptied and sediments analysed for total mass, particle size distribution and selected metal concentrations by six size fractions. The results of this sampling campaign are compared with the results of a 2005 study of the same gully pots to identify changes in the physicochemical properties of sediments over time and examine whether changes identified can be linked to changes in wider catchment management practices. The results highlight the potential impacts of winter road maintenance operations (e.g. up to a 15-fold higher solids loading rate in road catchment gully pots), reaching a normalised solids accumulation rate of 0.176–0.819 kg m^2^ year^−1^. An increase in tyre and road wear associated with winter road maintenance operations is also understood to contribute to the temporal increase of several metals including Cu, Zn, Co, Cr and V in the < 63-µm solids fraction in the road catchment gully pots. The concentrations of As and Pb decrease in all size fractions in both catchments, with the implementation of unleaded fuels (for Pb in housing catchment only), End-of-Life Vehicle Directive (Directive 2000/53/EC) (for Pb in both catchments), and strengthened industrial emission reduction measures suggested as possible drivers. The high contamination load for Zn, Cu, Cd and Pb in < 63-µm sediments from low-traffic housing catchment also emphasised the necessity of tracing and restricting non-traffic-related metal sources. Further seasonal monitoring of gully pot sediments is recommended to fully follow up the development of metals loading in both catchments.

**Supplementary Information:**

The online version contains supplementary material available at 10.1007/s11356-022-20341-8.

## Introduction

Mitigating diffuse pollution is identified in the EU Water Framework Directive (EU WFD 2000) as a measure to achieve good chemical and ecological status of water bodies. This objective also aligns with several UN Sustainable Development Goals (SDGs) including SDGs 6 (Clean water and sanitation), 11 (Sustainable cities and communities) and 14 (Life below water). Urban runoff is recognised as a major pathway for transporting diffuse pollutants from diverse sources to receiving waters (see Müller et al. (2020) for a comprehensive overview).

Approaches to mitigating urban diffuse pollution can be broadly categorised into two types: structural measures (e.g. stormwater quality-control facilities) and non-structural policies and practices which target pollutants at sources (e.g. substitution; eco-labelling; street sweeping). Stormwater quality-control facilities are often regarded as an end-of-pipe solution and can be applied at different (sub-) catchment scales. Regional-scale solutions include larger storage systems, e.g. stormwater ponds that receive the runoff from a whole catchment of multiple land-use types. Local-scale solutions include swales, rain-gardens, green roofs and gully pots (GPs) which drain from a relatively smaller area. Whilst end-of-pipe solutions have shown their efficacy in alleviating the adverse impacts of urban runoff quality on receiving waters, increasing attention is focusing on opportunities to mitigate the release of diffuse pollutants at sources as upstream, cost-effective policy-based control measures (Urbona 1998). However, the development of such source control policies requires a robust, comprehensive evidence base of, for example, inventory of diffuse pollutant sources and their release patterns, mobilisation and behaviour in urban drainage systems (Holtz 2006). Successful examples to-date include phasing out Pb in gasoline (Viklander and Marsalek 2011) and concerted efforts in removing polychlorinated biphenyls (PCBs) from Swedish buildings (Bernevi Rex 2019). However, approaches to fully evaluate the efficacy of these policies remain an open challenge.

Examples of directives addressing both point and diffuse pollutions include the European Union Water Framework Directive (EU WFD 2000); identifies substances for reduced emission (hazardous substances) and cessation (priority hazardous substances) to the environment), the EU Industrial Emissions Directive (IEF) (2010; enhanced requirements to reduce industrial emissions to air, soil and water) and the EU End-of-life Vehicles (ELV) Directive (2000; restricts the use of harmful substances). Whilst several studies have reported considerable decreases in the stocks of many substances within the urban technosphere e.g. Cd and Pb (Månsson et al. 2009), the impact of these broad-scale measures on urban diffuse pollution loads has yet to be robustly interrogated. This is of particular concern within a Swedish context, where soil and water systems are vulnerable to metal pollution due to the typically elevated levels of soil acidity, low levels of calcium and magnesium salts in inland surface waters and the low salinity of the surrounding Baltic sea (Parkman et al. 1998). In comparison to water samples which provide only a ‘snapshot’ of pollutant occurrence and concentrations, the analysis of sediments provides an opportunity to develop a time-integrated understanding of pollution at a site level (Birch et al. 2000). Building on the findings of an initial GPs study by Karlsson and Viklander (2008), this study explores and evaluates the temporal changes in selected metal concentrations associated with GPs sediments as a potential approach to evaluating the impact of mitigation measures applied at a catchment scale.

## Materials and methods

### Presentation of study sites

The eight GPs are located in two types of catchments in Luleå, Sweden. Four GPs (R1–R4) are located along a highway with an annual average daily traffic (AADT) of over 10,000 vehicles/day (v/d), of which 8% are heavy goods vehicles. A further four GPs are located in a housing area (H1–H4) consisting of detached residences with chimneys (for wood-burning heating), private garages and gardens, with an AADT of less than 500 v/d (Swedish Transport Administration 2021).

### Sampling procedures

The current sampling programme took place 21^st^–25^th^ September 2020 (impossible to collect sediments from H1 as inadequate protection during road repaving works led to its infilling with asphalt). Full information on the dimensions, accumulation periods and contributing drainage areas of the seven GPs sampled is provided in Table 1. GPs investigated in this work have a volume of 80–104 L, which is similar to the UK design of 90 L (Butler and Memon 1999) and slightly larger than the Dutch design of 4–70 L (calculated using data from Post et al. (2016) and Rietveld et al. (2020b)). However, the Swedish, UK and Dutch designs are considerably smaller than the Swiss design of 250–450 L (Conradin 1989) and the Norwegian design of 790 L (Lindholm 2016).

**Table 1:** Basic information for the seven gully pots sampled by Karlsson and Viklander (2008) and the current study

	R1	R2			R3	R4		H2		H3		H4	
Sediment accumulation time in current study (year)	2	2			2	2		13		13		13	
Sediment accumulation time (year) reported by Karlsson and Viklander (2008)	1	0.3			0.3	0.3		> 25		> 25		> 25	
Inner diameter (mm)	500												
Inner surface area (m^2^)	0.19625												
Distance from road surface to the top of outlet (mm)	1770	1420			700	745		2000		2250		2010	
Sand trap depth (mm)	530	480			500	525		450		410		450	
Sand trap volume (L)	104	94			98	103		88		80		88	
Sediment bed thickness (mm)	420	505			630	675		430		430		270	
Volume of sediment bed (L)	82	99			124	132		84		84		53	
Presence of in-pot standing liquor	No												
Total solids dry mass (kg)	145.1	125.4			139.3	137.0		168.2		53.5		61.9	
Connected road surface area (m^2^)	413	97			85	187		280		310		430	
Connected roof surface area (m^2^)	/	/			/	/		20		150		120	
Directly connected impervious area (m^2^)	413	97			85	187		300		460		550	

The size of the directly connected area to each GP was determined by implementing a catchment delineation process in ArcGIS software-based on aerial photography-based digital elevation data (resolution of 0.25 m) for Luleå, provided by Läntmateriet (2021). The catchment delineation process can be summarised into the following key steps. The first step involves assigning a drainage capacity to each GP in the study area by converting GP surface gratings into circular polygons with a diameter of 500 mm and assigning an elevation level of − 10 m relative to the terrain surface. The second step involves implementing a flow direction estimation using the eight-direction flow approach proposed by Jenson and Domingue (1988), followed by the calculation of flow accumulation in each cell. The sub-catchment for each GP was then delineated by integrating step one and two outputs with GPs chosen as outlet points.

GPs were emptied by an eductor truck that employed hydraulic dredging to loosen the compacted sediment bed in combination with a vacuum pump to collect the sediment–water mixture. After each GP was emptied, the collected mixture (consisting of GP sediments, in-GP standing water and dredging water) was placed in a container lined with a layer of new high-density polyethylene (HDPE) to avoid contact between sample and container. The eductor truck was thoroughly cleaned between GPs to avoid cross-contamination between sites. The sediment–water mixture in each container was allowed to stand for 1 h before supernatant water samples were collected from each container and stored at 4 °C prior to the analyses. The remaining water volume was decanted using a peristaltic pump, and the total collected water volume was recorded. The container and wet slurry were then weighed before sediment samples were taken using the conning and quartering procedures described in ASTM (1985).

### Laboratory analyses

#### Laboratory analyses of water samples

Water samples were analysed for suspended solids (SS) and selected metals (As, Cd, Co, Cr, Cu, Ni, Pb, V and Zn) in both particulate and dissolved fractions (collected on and filtered through 0.45-µm membrane filters, respectively). SS was determined using the multiple filter method (MFM) proposed by Nordqvist et al. (2011) where the sample is vacuum filtered through three filters of decreasing pore sizes (25, 1.6 and 0.45 µm). Filters were weighed after drying at 105 °C. For dissolved fractions, aqueous samples were digested with 1 ml HNO_3_ for every 100 ml sample and then analysed with sector field inductively coupled plasma mass spectrometry (ICP-SFMS) following SS-EN ISO 17294–2:2016 and US EPA Method 200.8:1994. For particulate fractions, samples were dried at 50 °C and digested before analysis with ICP-SFMS following modified SS EN ISO 17294–1,2 and US EPA Method 200.8.

#### Laboratory analyses of sediment samples

Sediment samples were analysed to determine particle size distribution (PSD), dry mass fraction (f_d_) and selected metals. Mass-based PSD analyses followed ISO 11277:2009, where sediment samples were first wet-sieved to 63 µm with the < 63-µm size fraction further analysed using laser diffraction. Dry mass fractions are reported as the ratio of sample dry mass (residue weight after drying at 105 °C for 20 h, with sediments held in a desiccator until a constant weight) by the sample wet weight. Selected metals contents were determined for the total fraction (≤ 2 mm) and for six size fractions (< 63 µm, 63–125 µm, 125–250 µm, 250–500 µm, 500–1000 µm and 1000–2000 µm). These size fractions were derived by first drying sediments at 50 °C, followed by their sieving through woven stainless steel wire cloth sieves of corresponding pore sizes. Metals were determined by ICP-SFMS following modified SS-EN ISO 17294–1,2 and US EPA Method 200.8:1994, after being digested with 5 ml 7 M nitric acid in a modified microwave oven. A lower reporting limit for metals (Table 2) is provided in this work due to the use of ICP-SFMS in the current work.

**Table 2:** List of reporting limits for sediment metals in both studies

	Reporting limit (mg/kg)	
Metals	Karlsson and Viklander (2008)	Current work
As	3	0.1
Cd	0.1	0.01
Co	0.1	0.03
Cr	0.2	0.1
Cu	0.3	0.3
Ni	0.2	0.08
Pb	1	0.1
V	0.2	0.2
Zn	1	1

LoI was determined in identified size fractions following SS 028,113, where samples were dried at 105 °C for 20 h and thereafter in a muffle furnace at 550 °C for 2 h.

#### Laboratory analyses of winter road maintenance traction materials

Three types of typically applied traction grit materials for winter road maintenance in Luleå were also analysed to determine PSD. Identified as Material A (branded aggregated sizes of 2–6 mm), Material B (branded aggregated sizes of 4–8 mm) and Material C (branded aggregated sizes of 0–6 mm), Material A and B are used on residential streets, pedestrian and bike paths (including the H2-H4 study area), whilst Material C is used on high trafficked roads (including the road catchment in this study where R1–R4 are located).

### Data analysis

#### Contamination factor

Contamination factors (CF) for selected metals (As, Pb, Cd, Cr, Ni, Cu, Zn, V and Co) were calculated using local background soil concentration data (available for the < 63-µm fraction) reported by Andersson et al. (2014). CFs were calculated using Eq. (1):

1 $${\text{CF}}_{\text{catchment-year}}= \text{ } {\text{C}}_{\text{gully pot}}\text{/}{\text{B}}$$

where *CF*_catchment-year_ = contamination factor; ${\text{C}}_{\text{gully pot}}$, metal concentration for GPs sediment samples (< 63 µm) from individual GP; *B* = local background concentration in local native soils (< 63 µm).

#### Solids accumulation rate normalised by accumulation period and drainage area

The GPs investigated in this work and other studies do not share the same accumulation time period or receive runoff from the same sized catchment areas. As both parameters impact on the total mass of solid accumulated in GPs, the use of absolute values hinders the direct comparison of total solid dry mass between sites and studies. To address this, the total dry mass of solids in each investigated GP was normalised by the accumulation period and drainage catchment area, expressed as per Eq. (2):

2 $${\rm I}=(SS\cdot V+{f}_{d}\cdot {M}_{\mathrm{wet}} )/(T\cdot A)$$

where *I* (kg/(year·m^2^)) = normalised solids accumulation rate in GP; *SS* (mg/L) = suspended solids; *V* (L) = volume of discharged water; *f*_*d*_ = dry fraction; *M*_wet_ (kg) = mass of wet solids; *T* (year) = length of accumulation period; *A* (m^2^) = total effective connected area.

#### Statistical analyses

Anderson–Darling normality tests indicated a non-normal distribution of the dataset, and therefore non-parametric methods were used. Mood’s median tests (Minitab 19) were applied to examine the level of significance associated with (1) temporal changes of selected metals determined within the six sediment size fractions for each catchment type; and (2) impacts of catchment types on selected metal contents in sediments by size fractions for respective sampling occasion. Spearman’s rank-order correlation tests (Minitab 19) were also applied to examine the significance of relationships between selected metals in sediments by six size fractions for the respective sampling occasion. The correlation is considered strong if correlation coefficient (*ρ*) > 0.6 and significant if *p* < 0.05.

## Results

### Particle size distribution

#### Gully pot sediments particle size distribution

**Fig. 1:**
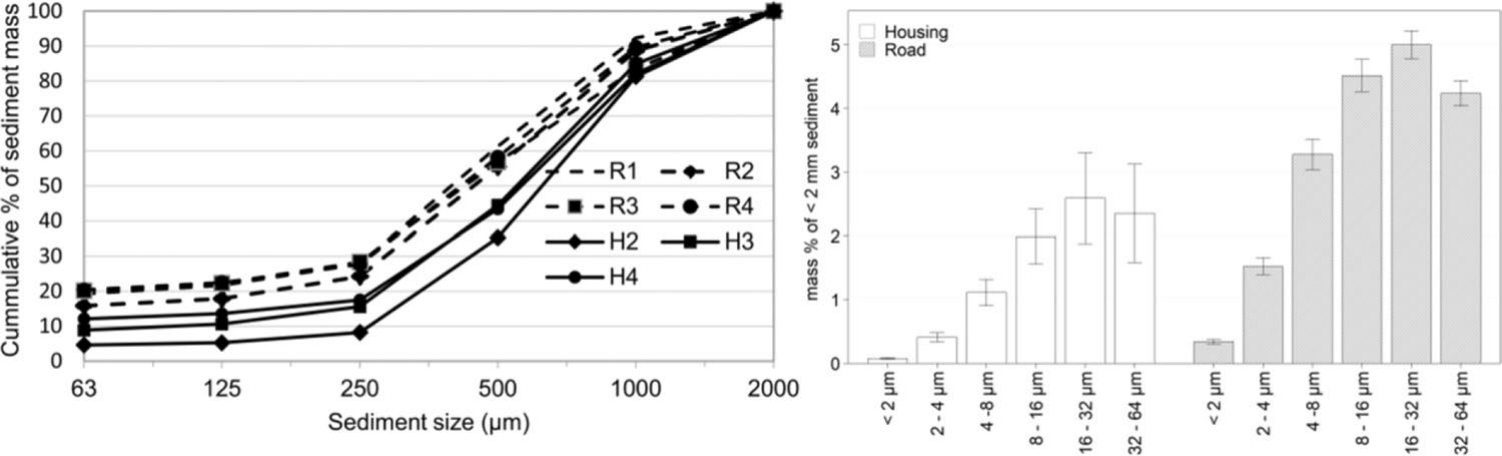
**A**: particle size distribution of gully pots sediments (63–2000 µm); **B**: particle size distribution of gully pots sediment < 63 µm by catchment types. Error bars represent the standard deviation of solids mass percentage for each size fraction

The GP-specific PSD curves for the current study are presented in Fig. 1. Overall, the PSD curves exhibit a clear difference by catchment type (Fig. 1A). Sediments from road catchment GPs (R1–R4) present a *d*_50_ of < 500 µm, and the sediments of R2 and H2–H4 present a *d*_50_ of > 500 µm. In regard to < 2-mm sediments, sediments from R1–R4 also consist of a higher percentage (a mean of 19% by mass) of < 63-µm particles than those from H2–H4 where particles in this size range account for less than 10% (by mass) of total sediments. For both catchments, particles within the size range of 8–63 µm dominate the < 63-µm sediments (Fig. 1B).

#### Traction grits particle size distribution

**Fig. 2:**
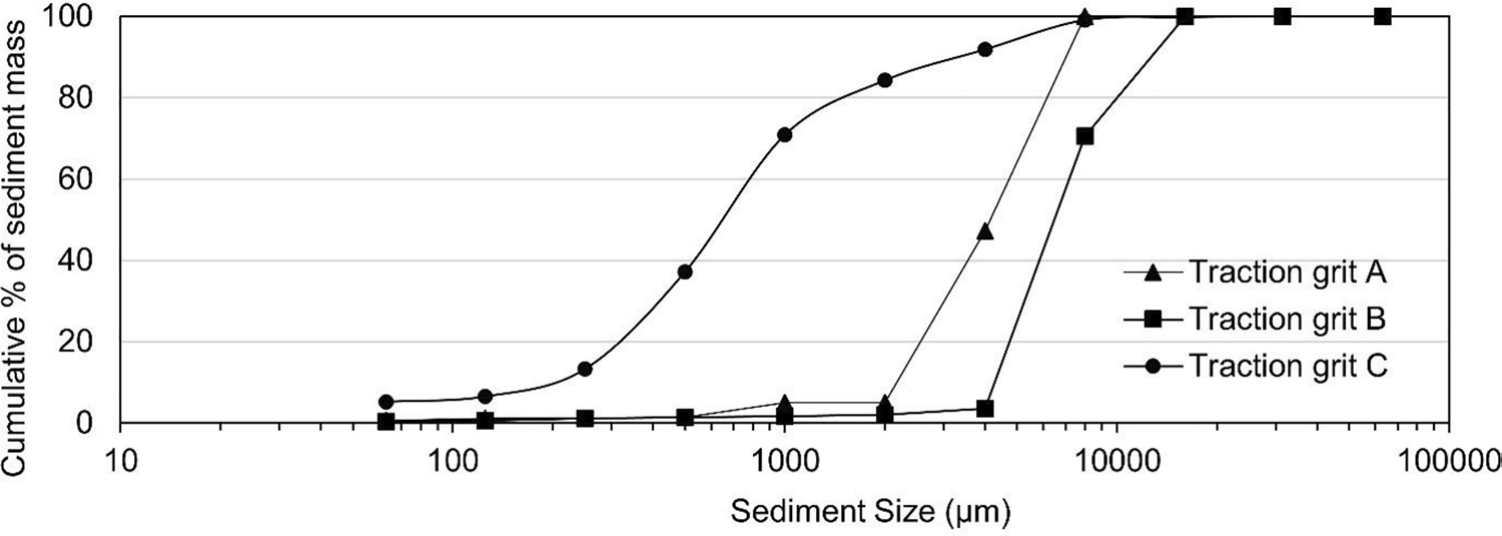
Particle size distribution curves for three types of traction grits used by the local municipality

Figure 2 presents the PSD curves for the three types of tractions grits that are used by the local municipality. Traction grits A, B and C exhibit a d_50_ of 5000 µm, 6000 µm and 650 µm.

### Normalised solids accumulation rate

**Fig. 3:**
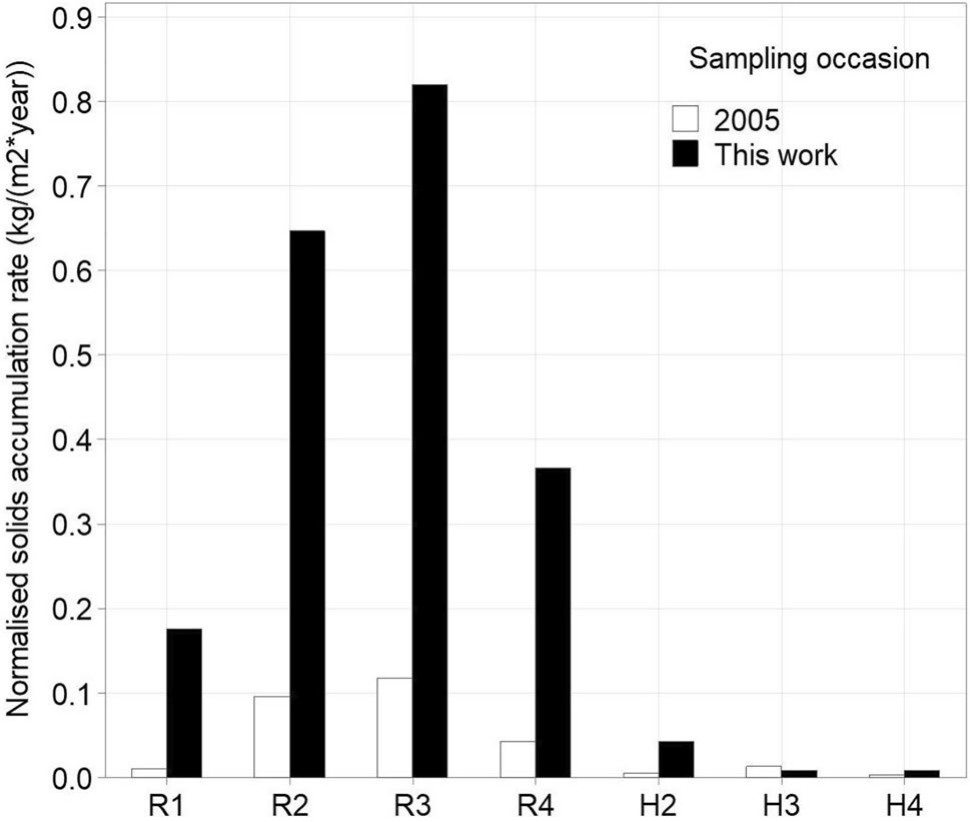
Normalised solids accumulation rates in gully pots for respective sampling occasions

Figure 3 presents the normalised solids accumulation rates by GP and sampling occasions. Both sampling occasions indicated a higher sediment accumulation rate in the road catchment than that of the housing catchment. Compared to the previous sampling by Karlsson and Viklander (2008), the normalised solids accumulation rates for road catchment GPs for the 2020 sampling also witnessed an approximately eightfold increase, reaching a mean value of 0.428 kg m^−2^ year^−1^ compared to that of 0.058 kg m^−2^ year^−1^ for the 2005 sampling campaign. A similar level of increase of normalised solids accumulation rate is not reported for the housing catchment GPs. In both campaigns, the normalised solids accumulation rate followed a similar pattern of R3 > R2 > R4 > R1.

### Concentrations of selected metals by size fractions

**Fig. 4:**
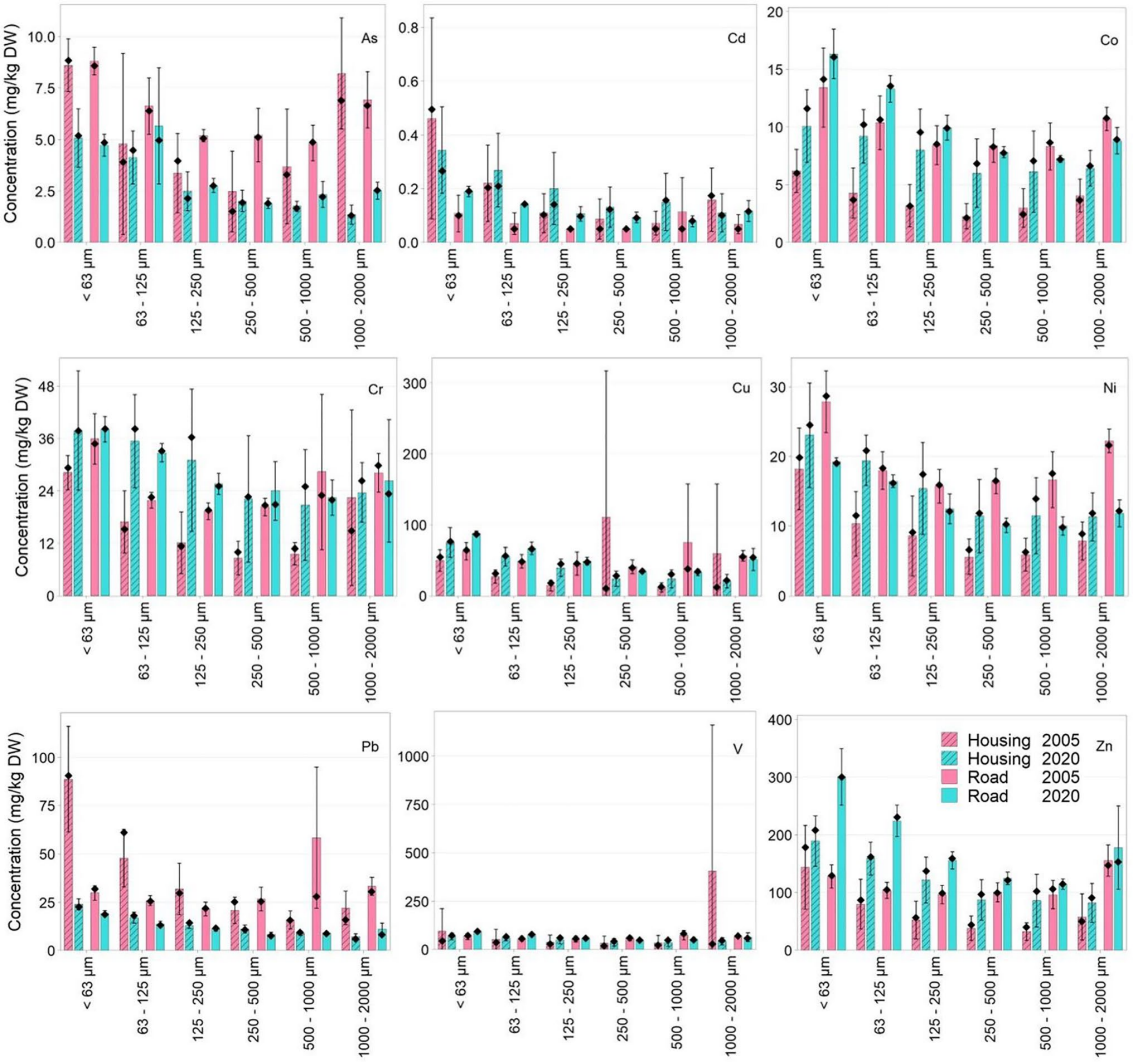
Concentrations of As, Pb, Cd, Zn, Cr, Cu, Co, Ni and V by size fraction in gully pots sediments collected on two sampling occasions. Bars denote the mean concentrations for metals of respective size fraction, catchment type and sampling occasion. Black dots denote the median concentrations. Error bars denote the standard deviations

Figure 4 presents the metal concentration per size fraction per metal for both the 2005 and 2020 sampling occasions. Median concentrations of Pb and As show a decrease in concentration between the occasions for all size fractions (*p* < 0.05) from the road catchment (except 125–250 µm and 500–1000 µm) and only the < 63 µm and 1–2-mm fractions in the housing catchment (Figs. S3 and S4). In contrast, concentrations of Cr, Cu, Zn, V and Co indicate an increase between the sampling occasions, especially for the < 250-µm sediment in both catchments (Fig. 4). However, a significant increase (*p* < 0.05) was only identified for the < 63-µm fraction from the road catchment and the 63–125-µm sediments from the housing catchment. Cd also exhibits an overall increase for both catchments (insufficient data for statistical analysis as over 50% of Cd concentration were below the reporting limit in Karlsson and Viklander (2008). In contrast, changes in Ni concentrations between sampling campaigns are not consistent between catchments, with a decreasing trend observed for road catchment sediments (at a significant level for the < 63 µm, 250–500 µm and 1–2-mm sediment fractions) and an increasing trend for housing catchment sediments (significant increase for the 63–125-µm sediment fraction).

### Contamination factor

**Fig. 5:**
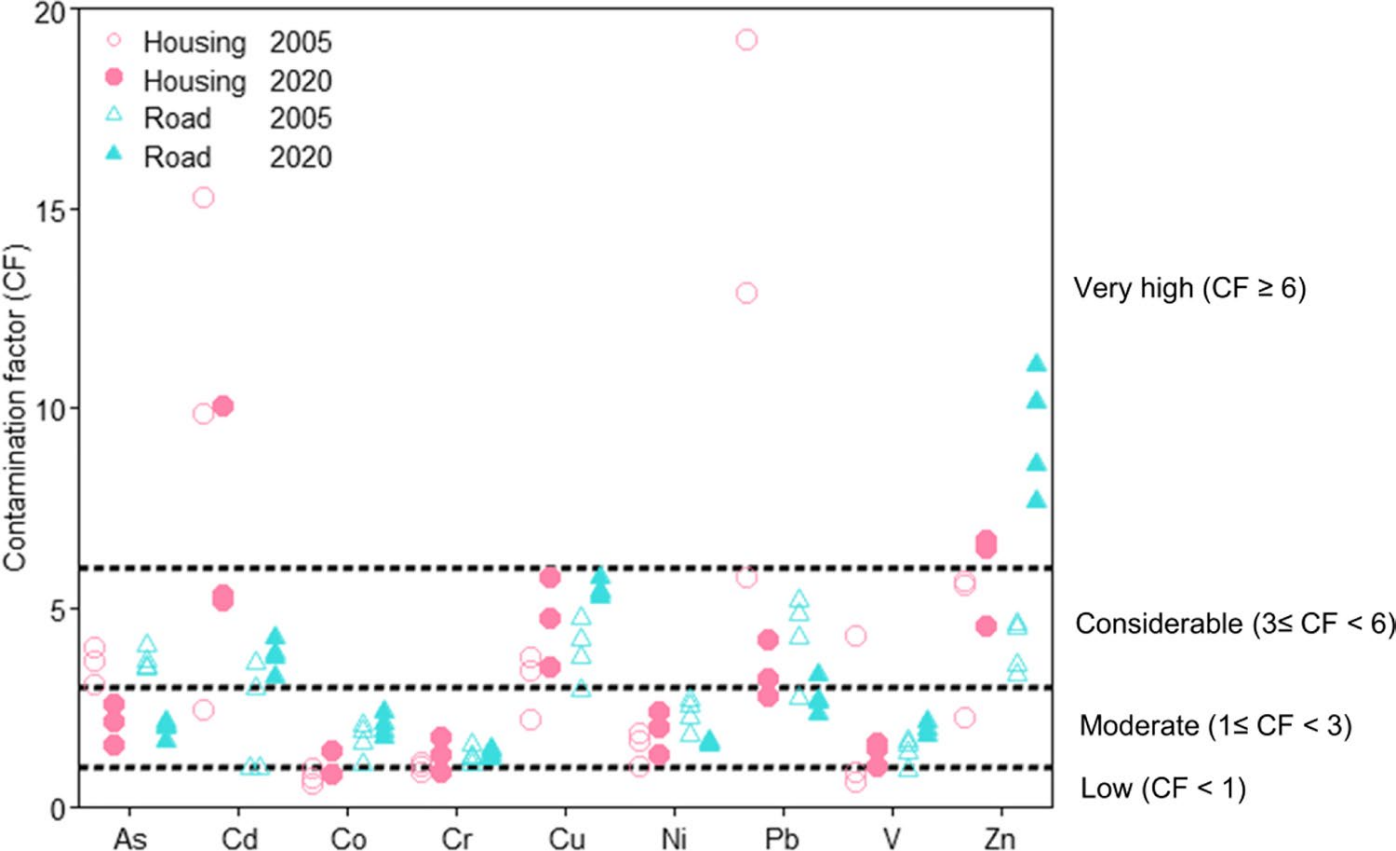
Contamination factors (CF) for gully pot sediment samples (< 63 µm) by catchment types and sampling occasions (dashed lines refer to CF categories developed by Hakanson (1980))

Contamination factors (CF) are used to quantify the magnitude of soil/sediment contamination relative to values reported for local uncontaminated soil. Figure 5 presents CF for selected metals by catchment type and sampling occasions. Principally, GP sediments are classified as at least moderately contaminated (except for Co, Cr and V in selected housing catchment sediments which are classified as low), evidencing the impact of anthropogenic activities on the load of metals carried by sediments in both catchments during both campaigns.

CF for both Cu and Zn showed a clear increase in both catchments between the campaigns. For example, in the road catchment, the Zn CF more than doubled from a mean of 4.01 for the 2005 campaign to 9.39 in the latest sampling occasion (resulting in a classification of very high) and Cu increased from a mean CF of 3.92 in the 2005 campaign to 5.46 in the 2020 sampling occasion. Though the CF of Zn and Cu did not exhibit an equivalent level of increase in housing catchment sediments, an increase in CF was detected and sediments were classified as (or close to) a very high contamination level.

In terms of Cd, whilst the road catchment sediments increased from a mean value of 2.16 to 3.79 (a shift from a moderate to a considerable contamination level) between sampling campaigns, the trend in the housing catchment is less clear. This is exemplified with the Cd CFs of housing catchment sediments in the 2005 campaign demonstrating significant levels of variation from moderate to very high levels. A similar pattern in Cd CFs is also shown in the 2020 sampling campaign, suggesting that activities at an individual housing (or plot level) have the potential to generate localised pollutant hotpots. A similar range in CF is also shown for Pb in the same 2005 housing catchment GP sediments, supporting the potential for residential activities to impact at a localised scale. However, a similar range in Cd and Pb levels is not seen in the housing catchment 2020 data set or observed in the road catchment data set where, overall, Pb concentrations showed a decreasing CF over time.

## Discussions

The presented physicochemical properties were not reported for sediments from a specific year/time point but represent the physicochemical characteristics of a time-composite sediment sample of the entire sediment bed during the whole accumulation period prior to the sampling occasion. The physicochemical composition of GP sediments is a function of the activities which take place in the catchment between GP emptying campaigns. The following sections consider GP sediment composition data in relation to catchment activities with a focus on traffic and related winter road maintenance operations, regulatory changes and the influence of non-traffic sources.

### Impacts of traffic and related winter road maintenance operations

**Fig. 6:**
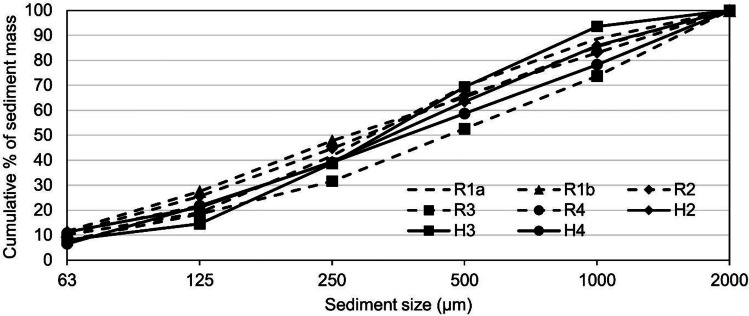
Particle size distribution of sediment (63–2000 µm) reported by Karlsson and Viklander (2008). The R1 gully pot was emptied twice in the work by Karlsson and Viklander (2008), where R1a represents sediments accumulated over 5 years and R1b represents sediments accumulated in 1 year

Compared with the PSD of GP sediments derived from the 2020 sampling campaign (Fig. 1), those collected by Karlsson and Viklander (2008) did not exhibit a clear catchment-type-dependent pattern (Fig. 6) though a consistently higher traffic volume (and proportion of heavy goods vehicles) are reported for the road catchment (relative to the housing catchment) during both sampling campaigns (Swedish Transport Administration, 2021).

Comparing the PSD curves from both sampling campaigns, a threefold higher mass percentage of < 63-µm sediments fraction was reported in road catchment GPs in comparison with the previous sampling occasion (Figs. 1 and 6). The identified PSD pattern change is not related to the traffic volume as (noted above) the reported AADT did not exhibit a major change over the past two decades in either catchment (Luleå municipality, pers. com). Instead, winter road maintenance operations (e.g. spreading of traction grits, snow ploughing and the mandatory requirement to equip vehicles with studded tyres) may be a contributing factor to the higher identified mass fraction of < 63-µm size fraction sediments, given that the sediment accumulation period for 2005 (R2–R4) sampling campaign did not include a winter-spring season. Winter road maintenance operations intensify levels of tyre and road wears (Lindgren 1996), but also introduce further abrasion opportunities between tyre studs and traction grits leading to an increased generation of fine particles (Viklander 1998). Whilst winter road maintenance activities are also undertaken in the housing catchment, their contribution to the generation of fine particles is not of the same magnitude as in the road catchment, due to a lower AADT, less frequent snow ploughing (less road surface abrasions) and the use of larger-sized traction grits (traction grit A and B is used in housing catchments; C is used for the road catchment) (Fig. 2).

Both the 2005 and 2020 sampling campaigns indicate higher normalised solid accumulation rates in the road catchment in comparison to the housing catchment (Fig. 3). Such a feature aligns with the fact that more intensive traffic activities (both higher AADT and a higher proportion of heavy vehicles) in the road catchment give rise to a higher solids supply from a range of sources including fuel combustion and brake, tyre and road wear (e.g. Boulter et al. 2006; Klein et al. 2021). The release of wear-derived particulates is also expected to be further enhanced by winter road safety measures (Carlsson et al. 1995; Boulter et al. 2006), which may also contribute to the increase in solids loading rates in road catchment GPs in the 2020 sampling campaign which included the winter season (Fig. 3). For example, the municipality reports that during a normal winter ca. 16,000 tons of traction grits are applied within the city of which only 25% are typically collected during the late-spring street sweeping practices. With GPs being potentially one of the receptors for traction grits, the fact that GP sampling campaigns including winter season have three times the normalised solids accumulation rates in comparison to a GP sampling campaign that did not include a winter season indicates both a spatial and temporal relationship between GP contents and their local environment. Over recent decades, more frequent freeze–thaw cycles in Sweden have been reported (Nilsson et al. 2021), and such a tendency is projected to intensify under climate changes (Sarady and Sahlin 2016), leading to a possible increased use of traction materials to address winter road safety issues. This might additionally contribute to an even higher solids loading rates in GP system unless a more efficient traction grits collection technique is adopted. Compared to the solids loading rates reported in the literature (Table 3), the values reported for the road catchment GPs of this study are much higher (though within the same order of magnitude) as those previously reported for the same catchment (Karlsson and Viklander 2008).

**Table 3:** Reported time-averaged solids loading rate in literatures and the current study

Catchment type	Solids loading rate (kg/(m^2^ year))	Measurement method	Reference
Residential	0.009–0.043	Fully emptying GPs	Current study
Trafficked road	0.176–0.819		
Highway	0.001168–0.04015	^1^Nylon net at inlet	Ellis and Harrop (1984)
Trafficked road and residential	0.086–0.093	Fully emptying GPs	Grottker (1990)
Residential	0.004–0.014	Fully emptying GPs	Karlsson and Viklander (2008)
Trafficked road	0.011–0.118		
Residential	0.00186–0.1095	^2^Nylon net at inlet	Rietveld et al. (2021)

Key: 1 & 2: Nylon nets with a minimum pore size of 63 µm and 50 µm (for respective study) were placed at the inlets of GPs to capture inflowing solids

Besides the catchment differences related to the availability of solids being supplied to the investigated GPs, the differences in the GP designs may also contribute to the varied solids accumulation rates between sites. GPs in the road catchment have generally deeper sumps compared with those of housing catchment GPs (Table 1). Such a difference may result in a higher retention efficiency in road catchment GPs, as was suggested by both Lager et al. (1977) and Rietveld et al. (2020a). Also, the GPs in the housing catchment have higher distances from the road surface to the top of outlet compared with that of road catchment GPs (Table 1), resulting in a potentially higher kinetic energy of the impinging runoff induced by gravity. The higher energy head of the inflow runoff may also inhibit retention efficiency. However, due to the sample size and scope of this work, no further evaluation of this hypothesis could be undertaken within the current study.

As described in Sect. 2.4.2, the normalised solids accumulation rates presented in this work do not assume a constant sediments accumulation behaviour over the monitored time-frames, but instead account for differences in accumulation time periods and catchment sizes. The achievement of a hypothetically constant sediments accumulation rate would require a steady solids supply to the drainage catchment, stationary transport of solids to GPs in both wet and dry weathers and uniform solids retention efficiency, all of which are thought unlikely given the inherent variability of processes within am urban catchment. This is demonstrated by the results presented by Post et al. (2016), where sediment bed levels in 95% of 300 monitored GPs already stabilised after 4–5 months of the 15-month monitoring period. This finding is potentially attributed to the decreasing retention efficiency by the raising sediment bed levels (Post et al. 2016) and the intensified scouring processes by the decreasing standing water depth (Avila 2008) with the stabilised sediment beds being an indication of an equilibrium status of both in-pot processes.

Based on the findings of Post et al. (2016), it is also plausible to suspect that sediment beds of the housing catchment GPs had reached an equilibrium status long before both sampling occasions, and hence normalising the solids dry mass by the entire accumulation period would effectively be an under-estimation of field conditions. However, even if the accumulation period for the housing catchment GPs is shortened to 2 years (i.e. the same as the road catchment GPs), the mean normalised solids accumulation rate for housing catchment GPs remains almost four times lower than that of road catchment GPs, suggesting the observed difference by catchment is not entirely due to the time-induced bias but also other factors including above mentioned solid source differences and GP designs. This still highlights the necessity of GPs emptying in maintaining their treatment functions rather than simply targeting GPs from filling completely and further causing flooding issues, noting that sediment beds in only 5% of the 300 monitored GPs by Post et al. (2016) reached the outlet level over the 15-month time-frame. Further, blocked GPs do not necessarily lead to flooding as adjacent GPs—if well-functioning—would reduce the exposure to such risks as suggested (Post et al. 2016).

With regard to changes in metal concentrations (Fig. 4), the limited changes in AADT over the past two decades in either catchment (Luleå municipality, per. Com) contributes to the evidence base that traffic is only one source of metals within the catchments. A further (and less well understood) source of metals is traffic-related winter road maintenance operations which—by definition—have a seasonal impact on GP processes. As discussed above, similar types of winter road maintenance operations are applied in both catchments (though at differing intensities of e.g. application frequency) and are likely to affect the metal loading associated with sediments retained in GPs. For example, just as the application of traction materials exacerbates the wear and tear of tyres, tyre studs and the road surface (Carlsson et al. 1995), these type and amount of metals associated with GP sediments are also influenced. Metals that characterise brake and tyre wear such as Cu and Zn (McKenzie et al. 2009) are significantly increased in the < 63-µm fraction of the road catchment in the 2020 campaign, with the observed strong correlation between Cu–Zn (*ρ* = 1, *p* < 0.0005) indicating they derive from a common source. The impacts of winter road maintenance operations are also indicated by the strong correlations for Zn–V (*ρ* = 0.857, *p* = 0.014), suspended solids; *ρ* = 0.964, *p* < 0.0005 for < 63-µm fraction) and Zn-Co (*ρ* = 1, *p* < 0.0005 for < 63-µm fraction) in samples from the 2020 sampling, which are attributed to both the wear of V–Co-alloyed steel studs and/or the abrasion of V-containing asphalt (Johansson et al. 2009). However, a similarly strong correlation was not observed in sediments from the previous sampling event for the same size fraction again understood to be a function of the shorter accumulation time-frame which did not include a winter season.

A further factor to consider is the impacts of traction grits themselves on GP sediment metal concentrations. The concentrations of Pb, Cd, Zn, Cu, Cr, Co, V and Ni in unused traction grits applied to the Luleå catchment (Gavrić et al. 2021) are presented together with GP sediment total metal concentrations (Fig. S5). This indicates that traction gits contain elevated levels of a range of metals which could contribute to elevated metal concentrations and—for metals which report a relatively lower grit concentration—effectively ‘dilute’ concentrations of, e.g. Pb, Cd, Zn and Co. However, the exact quantification of the above enhancing/diluting impacts requires a fuller understanding of fractions GP sediments composed of traction grits.

The impact of grit materials on GP sediments from the housing catchment is less clear, due to the lack of metals data for traction grits used in the 2005 study but also the fact that grit materials (4–8 mm) used in the housing catchment are of a larger size fraction than those analysed (i.e. < 2 mm) for the determination of total metal concentrations. However, the presence of metals in road grits at concentrations higher than those reported in GPs sediments suggests that abraded grit particles may contribute to concentrations of Cr, Co, V and Ni (Fig. S5). With the total concentration of these metals being much higher in grit B than grit C, the abraded grit particles might also mask differences by catchment types.

The concentrations of Zn for < 63-µm sediments of the road catchment GPs in this study are at the same magnitude as the mean concentration of 395 mg/kg reported in Pitt (1985) for GP sediments of the same size fraction but more than three times lower than the mean concentration of 954 mg/kg reported for < 62.5-µm sediments by Birch and Scollen (2003), where the investigated GPs received runoff from streets with a traffic volume of up to 73,177 vehicles/day. In the same referred study, a mean Cu concentration of 181 mg/kg was reported for < 62.5-µm sediments, which was more than twice higher of that reported for < 63-µm sediments from road catchment GPs of the latest sampling occasion, indicating the influence of catchment specific sources on GP sediment quality.

### Impacts of regulatory changes

Whilst AADT remained relatively constant between sampling periods, a range of regulatory measures directly affecting catchment activities were implemented. For example, legislation requiring the removal of Pb additives from gasoline (Ordinance in SFS1985:838) was implemented in January 1995. This regulatory measure is particularly relevant to the decreases in Pb CFs in the housing catchment sediments between the two sampling occasions (Fig. 5) where the GP sediments from the housing catchment collected by Karlsson and Viklander (2008) had an accumulation period of over 25 years and could include sediments derived from pre-ban on Pb additives in fuel. However, decreasing Pb in road catchment sediments must have another explanation as the sediment accumulation of the previous sampling occasion commenced post-2005. The decrease of Pb in road catchment sediments could be attributed to further regulatory measures such as the ban on the use of Pb wheel weights and requirements to limit the contents of both Pb and Cd in brake linings. The initial implementation of this measure in Sweden began in 2003 (Swedish Ordinance (2003: 208)) as part of the implementation of the EU ELV Directive (2000) at a national level. The efficacy of the above measures was highlighted in SEPA (2021a) which reported a > 99% decrease in Pb emissions from the transport sector since 1990 (national wide). For Luleå alone, the annual total Pb air emissions from the transport sector were decreased by > 98% from 2.637 tons in 1990 to 0.0416 tons in 2018 (SEPA 2021b). The Pb concentrations for the finest (< 63 µm) GP sediments from both sampling occasions are considerably lower than the reported mean concentration of 780 mg/kg for < 62.5-µm GP sediments by Birch and Scollen (2003) and mean concentration of 1170 and 1970 mg/kg (two catchments) reported for < 63-µm GP sediments by Pitt (1985). Such a discrepancy is attributed to (1) sampling works of both referred studies were carried out by the full ban on leaded fuels and (2) large traffic difference among studies.

Though the implementation of the EU ELV Directive (2000) also limited the contents of Cd in automobiles, it only led to a marginal reduction in emissions from the Swedish transport sector. According to the SEPA (2021a), the emissions of Cd from road traffic have been almost stable since 1990, accounting for less than 1% (5 kg/year/Sweden) of the total annual Cd emissions in 2019. Compared with the 2005 sampling occasion, the mean Cd concentrations of the < 63-µm sediments from the road catchment GPs collected in 2020 showed a marginal increase from 0.166 mg/kg (*n* = 2) to 0.1895 mg/kg (*n* = 4).

### Impacts of non-traffic sources

In addition to the direct traffic-related sources, atmospheric deposition also contributes metals to the urban drainage system (e.g. Davis et al. 2001; Sabin et al. 2005; Gunawardena et al. 2013). The term atmospheric deposition refers to both short and long-distance transport processes though no definitive definition of either term has yet been agreed upon. In regard to the ‘short distance transport’, a direct example can be given by, e.g. Meland et al. (2010) where the enrichment of metals in the tunnel inner surface wash water was attributed to sources including combustions, brake, tire and road wear. This work emphasised the emissions from traffic activities did not necessarily directly accumulate on the ground but could be further transported to and deposited elsewhere. In terms of long-range atmospheric transport processes, several metals such as Pb, As and Cd are documented as transported by such processes but further descriptors of relevant processes etc. are not given (Johansson and Tyler 2001). Hence, the current understanding of the relative importance of contributing sources (i.e. local traffic vs short/long-range transport processes) is limited. This knowledge gap has implications for both source tracking (challenge of linking pollutants to potential release sites) and the development of programmes of measures (focused on achieving good ecological status). Despite the identified challenges, the dataset derived from the both sampling occasions still suggested a potential link between the changes in the non-traffic sources and identified temporal changes of metal contents in GP sediments.

Pb for example, followed by the implementation of e.g. the ban on Pb in fuel and the ELV Vehicles Directive, the contribution of this sector to the total annual atmospheric Pb emission in Luleå has seen a considerable decrease from 65.7% in 1990 to 6.4% in 2019 (SEPA 2021b). The emission reduction from the traffic sector led to the increasing proportion of atmospheric emissions from the industry and energy sector, reaching 68.5% of total annual atmospheric emission in Luleå in 2019 (SEPA 2021b). To proceed with improving the urban environment, great efforts have also been implemented in industries through e.g. improving purification equipment and reducing the use of fossil fuels. Such measures successfully contributed to an 86% and 32% reduction of industrial atmospheric Pb emission in Luleå during 1990–2019 and 2005–2019 (SEPA 2021b), which also acted as an additional driving factor for the decreasing Pb concentrations in GP sediments. A strong and significant correlation for As–Pb (*ρ* = 0.964, *p* < 0.0005) was identified for suspended solids of the 2020 sampling occasion, suggesting a potential common source for both metals. Though commonly reported as a traffic-related pollutants released through the fossil fuel combustion process (Ozaki et al. 2004; Shi et al. 2012), the contribution of As from the traffic sector in the urban catchment is considered minor according to SEPA (2008) with an estimation made that 50% of the As loading in urban streets originated from long-range transport processes. Referring to the emission record over the years 1990–2019, the traffic sector has never been identified as a major contributor (< 3% of total annual atmospheric As emission in Luleå). Instead, emissions from the local industry (72–41% of annual emissions during 1990–2019) as well as electricity and heating production (24–55% from 1990 to 2019) are reported to be more important sources of As in the urban catchment (SEPA 2021b). Similar to Pb, the augmented atmospheric emission control by the local industries reduced the annual atmospheric As emission by over 70% during 1990–2019, one of the dominant drivers for the reported > 50% total annual atmospheric As emission in Luleå during the same period (SEPA 2021b).

Cd is another metal that exhibits a strong and significant correlation (*ρ* > 0.6, *p* < 0.05) with Pb in suspended solids and sediment samples supporting earlier studies of roadside soil samples (Hjortenkrans et al. 2006) and swale soil samples (Gavrić et al. 2021). The common sources for Pb–Cd include fuel combustion and brake linings (McKenzie et al. 2009). Despite a considerably higher AADT in the road catchment (than the housing catchment), no significant difference in sediment Cd concentrations by catchments was detected for either sampling occasion. This agrees with the findings by Hjortenkrans et al. (2006) and Pun et al. (2019), where varied driving habits in association with street layouts were identified as a more important factor for the Cd emission than AADT. Of the road-catchment GPs, R2–R4 are located along a straight-driving road without needing of varying driving behaviours (braking, acceleration and deceleration). Even though R1 drains from a bus station where frequent ‘stop and go’ takes place, the sediment Cd concentration from R1 is at the same magnitude as the sediment Cd concentrations from other road catchment GPs. The annual atmospheric Cd emission from the domestic traffic sector (35.1 g/year; accounts for 0.14% of total atmospheric emissions in Luleå 2019) (SEPA 2021b) is much lower than that reported for Cd emissions to air from local industries (23.2 kg/year). On the contrary to the downtrend emission of Pb and As from local industries, the industrial emission of Cd displayed a sharp increase (> 1600%) during 2015–2019 due to e.g. expansion of the industry, which may have contributed to the overall elevated concentrations in both catchments (Fig. 4).

It is also worth mentioning the housing-catchment specific activities which may have additionally contributed to pollutants loading. For example, street car washing by local households may discharge loads of organic (e.g. polycyclic aromatic hydrocarbons and aliphatic) and inorganic (e.g. metals) pollutants to the urban drainage system in the catchment (Hedenmark and Alkeblad 2016). A survey made in this housing area in September 2007 showed that street car washing was still much more preferred to car washing at designated facilities (Olofsson 2009). However, results of the same survey also identified that households tried to eliminate the negative impacts on the environment by washing their cars in their private garden so that wash water would infiltrate into the soil, rather than draining the wash wastewater directly into GPs. Another potential source of metals in the housing catchment comes from the building materials such as painted wood façades (source of Zn, Cu, Pb and Cd; Davis et al. (2001)) as well as cement tile (V and Cr) and bitumen felt (Pb, Zn and Cu) roofing (Andersson-Wikström 2015).

## Conclusions

This study evaluated sediments collected from the gully pots during two sampling campaigns with a time interval of 15 years. The sediments derived from these two sampling occasions represented varying accumulation periods with data used to evaluate changes in normalised solids accumulation rates, particle size distributions and selected metals concentrations. Results are discussed in relation to changes within the catchment structural and non-structural activities. Results suggest that:

- Winter road maintenance operations e.g. applying traction grits and the compulsory use of studded tyres may contribute up to a 15-times higher solids loading rate (0.176–0.819 kg m^−2^ year^−1^) in road catchment gully pots of the latest sampling occasion than the previous one. These measures are also likely to have contributed to the higher mass fraction of < 63-µm particles in road catchment (mean of 19%) in comparison to the housing catchment (mean of 8.5%).
- Concentrations of As and Pb concentrations decrease in all particle size fractions in both catchments, with the implementation of the ELV Directive (Directive 2000/53/EC) (for Pb only) and strengthened industrial emission reduction measures (both As and Pb) implemented by local industries over the evaluated period suggested as possible factors in this change. Likewise, the relative increase in local industrial Cd emissions could contribute to the increase in Cd sediment concentrations in both catchments.
- A temporal increase of Cu, Zn, Co, Cr and V is observed, especially fine solids in both catchments. The increase in the road catchment concentrations is considered a function of winter road maintenance operations whilst the source of the temporal increase in the housing catchment remains unclear.
- CFs estimated for < 63-µm fraction indicate that the impact of anthropogenic activities can be identified at a GP level. The fact that there are high CFs for Zn, Cu and Cd in both catchments and Pb (housing catchment only) highlights that traffic is not the only source of metals.

By design, gully pot sediments reflect activities at a local catchment scale. However, the results of this study also suggest that GP pollutant concentrations may also be influenced by long-range transport processes. Hence, changes in GP sediment pollutant concentrations may also be used to evaluate the impact of wider catchment management practices. Further monitoring with an extension of the pollutants analysis list is therefore recommended not only to follow up the metals loading changes in both catchments but also to provide a basis for future identification of temporal changes to other pollutants of emerging concerns.

## Supplementary Information

Below is the link to the electronic supplementary material.

Supplementary file1 (DOCX 183 kb)

## Data Availability

All data generated or analysed during this study are included in this published article and its supplementary information files.
