# Supplementary material for: An evaluation of temporal changes in physicochemical properties of gully pot sediments
Source: Environ Sci Pollut Res Int. 2022 Apr 29;29(43):65452–65. doi: 10.1007/s11356-022-20341-8 (PMC9481490; doi:10.1007/s11356-022-20341-8)
Supplement: Supplementary file 1 — Supplementary file1 (DOCX 183 kb) [file 11356_2022_20341_MOESM1_ESM.docx]

# Supplementary Material

# Article title: An evaluation of temporal changes in physicochemical properties of gully pot sediments

Haoyu Wei^1^*, Tone Merete Muthanna^1,2^, Lian Lundy^1^ & Maria Viklander^1^

^1^Department of Civil, Environmental and Natural Resources Engineering, Luleå University of Technology, SE-97187, Luleå, Sweden

^2^Department of Civil and Environmental Engineering, Norwegian University of Science and Technology, NO-7491, Trondheim, Norway

*Corresponding author email: [haoyu.wei@ltu.se](mailto:haoyu.wei@ltu.se)

**Journal Name:** Environmental Science and Pollution Research

## Mood’s Median test

|  | < 63 µm | 63 - 125 µm | 125 - 250 µm | 250 - 500 µm | 500 - 1000 µm | 1000 - 2000 µm |
| --- | --- | --- | --- | --- | --- | --- |
| As |  |  |  |  |  |  |
| Pb |  |  |  |  |  |  |
| Cd | n/a | n/a | n/a | n/a | n/a | n/a |
| Cr |  |  |  |  |  |  |
| Cu |  |  |  |  |  |  |
| Zn |  |  |  |  |  |  |
| Ni |  |  |  |  |  |  |
| V |  |  |  |  |  |  |
| Co |  |  |  |  |  |  |

**Fig. S 1** Heat map for Mood’s median tests to detect if median metal concentrations by six size fractions statistically differ (p < 0.05) between sampling campaigns. This heat map is for sediment samples from road catchment only. Cells in dark green indicate a statistically significant temporal decrease of concentrations and cells in light green indicate an insignificant temporal decrease. Cells in dark red indicate a statistically significant increase of concentrations and cells in light red indicate an insignificant temporal decrease

|  | < 63 µm | 63 - 125 µm | 125 - 250 µm | 250 - 500 µm | 500 - 1000 µm | 1000 - 2000 µm |
| --- | --- | --- | --- | --- | --- | --- |
| As |  |  |  |  |  |  |
| Pb |  |  |  |  |  |  |
| Cd | n/a | n/a | n/a | n/a | n/a | n/a |
| Cr |  |  |  |  |  |  |
| Cu |  |  |  |  |  |  |
| Zn |  |  |  |  |  |  |
| Ni |  |  |  |  |  |  |
| V |  |  |  |  |  |  |
| Co |  |  |  |  |  |  |

**Fig. S 2** Heat map for Mood’s median tests to detect if metal median concentrations by six size fractions statistically differ (p < 0.05) temporally. This heat map is for sediment samples from housing catchment only. Cells in dark green indicate a statistically significant temporal decrease of concentrations and cells in light green indicate an insignificant temporal decrease. Cells in dark red indicate a statistically significant increase of concentrations and cells in light red indicate an insignificant temporal decrease

|  | < 63 µm | 63 - 125 µm | 125 - 250 µm | 250 - 500 µm | 500 - 1000 µm | 1000 - 2000 µm |
| --- | --- | --- | --- | --- | --- | --- |
| As |  |  |  |  |  |  |
| Pb |  |  |  |  |  |  |
| Cd | n/a | n/a | n/a | n/a | n/a | n/a |
| Cr |  |  |  |  |  |  |
| Cu |  |  |  |  |  |  |
| Zn |  |  |  |  |  |  |
| Ni |  |  |  |  |  |  |
| V |  |  |  |  |  |  |
| Co |  |  |  |  |  |  |

**Fig. S 3** Heat map for Mood's median tests to detect if median metal concentrations by six size fractions statistically differ by catchment types. This heat map is for sediment samples collected by Karlsson and Viklander (2008). Blue-shaded cells indicate significantly higher concentrations in road catchment

|  | < 63 µm | 63 - 125 µm | 125 - 250 µm | 250 - 500 µm | 500 - 1000 µm | 1000 - 2000 µm |
| --- | --- | --- | --- | --- | --- | --- |
| As |  |  |  |  |  |  |
| Pb |  |  |  |  |  |  |
| Cd |  |  |  |  |  |  |
| Cr |  |  |  |  |  |  |
| Cu |  |  |  |  |  |  |
| Zn |  |  |  |  |  |  |
| Ni |  |  |  |  |  |  |
| V |  |  |  |  |  |  |
| Co |  |  |  |  |  |  |
|  |  |  |  |  |  |  |

**Fig. S 4** Heat map for Mood's median tests to detect if metal median concentrations by six size fractions statistically differ by catchment types. This heat map is for sediment samples collected for the current study. Blue-shaded cells indicate significantly higher concentrations in road catchment and red-shaded cells indicate significantly higher concentrations in housing catchment

## Total metals of gully pot sediments and traction grits


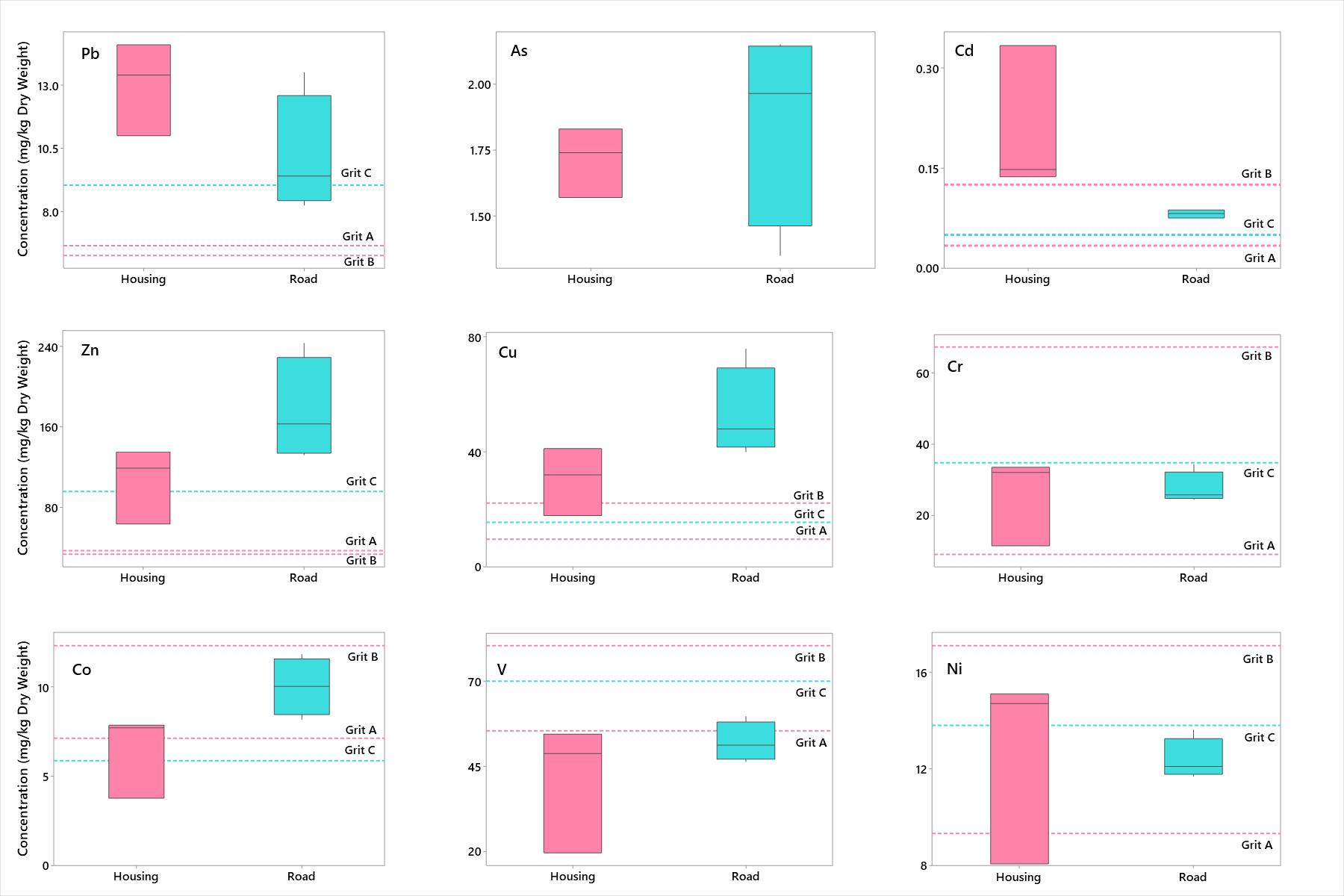


**Fig. S 5** Box plots for total concentrations of lead, arsenic, cadmium, zinc, copper, chromium, cobalt, vanadium and nickel in sediments (< 2 mm) collected in this study. Total concentrations of metals (except for arsenic) for traction grits materials applied in studied catchments were reported by Gavrić et al. (2021) and presented here as reference lines.
